# The impact of sleep disorders on brain network connectivity in postpartum women: a functional near-infrared spectroscopy-based study

**DOI:** 10.3389/fneur.2024.1487985

**Published:** 2024-12-12

**Authors:** Xia Chen, Shanguang Zhao, Danni Chen, Dan Zhang

**Affiliations:** ^1^Gynecology, Tsinghua University Hospital, Tsinghua University, Beijing, China; ^2^Department of Physical Education, Shanghai Maritime University, Shanghai, China; ^3^Department of Psychological and Cognitive Sciences, Tsinghua University, Beijing, China; ^4^Tsinghua Laboratory of Brain and Intelligence, Tsinghua University, Beijing, China

**Keywords:** postpartum depression, verbal fluency task, insomnia, functional connectivity, network topological properties, fNIRS

## Abstract

Sleep disorder is an important risk factor for postpartum depression. Although previous research has explored brain activity in postpartum depression, it has not fully revealed how insomnia affect mood by altering interactions between brain regions. This study aim to investigate the relationship between insomnia and depressive status in postpartum women, utilizing functional near-infrared spectroscopy (fNIRS) to explore brain network topological properties. Among 143 postpartum women, 40 were diagnosed with insomnia and 103 without. The results indicated that the Edinburgh Postnatal Depression Scale (EPDS) scores were significantly higher in the insomnia group compared to the control group. Compared with the control group, the insomnia group showed significantly increased connection strength of triangularis Broca's between middle and superior temporal gyrus and left between right dorsolateral prefrontal cortex (*p* < 0.001). Brain network topological analysis revealed that the small-world properties, clustering coefficient (*p* = 0.009), and local efficiency (*p* = 0.009) were significantly lower in the insomnia group compared to the control group. Notably, the local efficiency and clustering coefficient of the left temporal pole were significantly reduced and negatively correlated with EPDS scores. These findings elucidate how insomnia may exacerbate postpartum depression through changes in brain network properties. While the observed alterations in connectivity suggest a correlation, causation cannot be definitively established. Improving sleep quality remains a promising intervention, but further research is needed to clarify causal links and therapeutic targets.

## Introduction

Postpartum depression is a serious mental health issue that affects women after childbirth. Research indicates that ~10–15% of new mothers experience depressive symptoms following delivery, and sleep disturbances are considered one of the significant risk factors for postpartum depression (1, 2). The decline in sleep quality is strongly associated with the worsening of postpartum depressive symptoms, which not only affects the mother's emotional state but also has the potential to impact the quality of care provided to her family and newborn (3, 4). Although previous studies have acknowledged the crucial role of sleep problems in postpartum depression, the underlying neural mechanisms remain unclear. A deeper understanding of these mechanisms is essential for developing effective interventions.

Research has revealed the neural representations of postpartum depression and sleep disturbances, providing a biological foundation for understanding these complex psychological issues. Utilizing technologies such as electroencephalography (EEG), functional magnetic resonance imaging (fMRI), and functional near-infrared spectroscopy (fNIRS), studies have uncovered the neural activity patterns and underlying mechanisms of postpartum depression. Resting-state studies indicate that the default mode network (DMN) in postpartum depression patients exhibits abnormal activity, particularly with significantly weakened functional connectivity between the anterior cingulate cortex and the medial prefrontal cortex (5). Additionally, EEG studies have shown decreased alpha rhythms and increased beta rhythms in postpartum depressed women, indicating abnormal brain electrical activity during rest (6, 7). In task-based studies, the prefrontal cortex's functional connectivity shows significant abnormalities during emotion regulation tasks, further underscoring its critical role in emotional regulation and cognitive functions (8, 9). Similarly, studies on the neural representations of sleep disturbances have also revealed shared neural mechanisms associated with depression, particularly in the frontal cortex and brain network functions. fNIRS studies have found that sleep disturbances are closely related to changes in the oxygenation levels of the frontal cortex, with sleep deprivation leading to decreased oxyhemoglobin levels in the frontal cortex, consistent with declines in cognitive function and emotional regulation capabilities (10, 11). Like depression patients, those with sleep disturbances exhibit significantly reduced prefrontal cortex activation during tasks, such as in language generation and decision-making tasks (12). In task-based studies, EEG can capture rapid neural responses, such as event-related potential changes during emotional tasks (13), while fMRI can reveal abnormal functional connectivity between the prefrontal cortex and the amygdala (8, 9). For example, in verbal fluency tasks (VFT), studies have found that postpartum depression patients have significantly lower oxyhemoglobin levels in the prefrontal cortex compared to healthy controls, suggesting potential impairments in executive function and language generation (14). fNIRS has shown unique advantages in these task-based studies, such as portability and low sensitivity to motion artifacts (15). Additionally, research has indicated that the newborns of mothers with prenatal depression exhibit abnormal brain oxygenation levels and hemodynamic response disorders in the prefrontal cortex (PFC), which may affect their emotional and cognitive development (16).

Although substantial analyses have been conducted on brain region activity in postpartum depression, these studies have often focused solely on the activation of individual brain regions, neglecting the interactions between regions and their overall impact within the network structure. Particularly in research related to small-world properties associated with sleep disturbances, brain network analysis provides a more comprehensive approach to understanding the complexity of brain function. For instance, by analyzing the topological characteristics of brain networks, such as node degree, clustering coefficient, and small-worldness, researchers can identify brain regions and connectivity patterns that may play a critical role in patients with depression (17, 18). Studies have shown that the brain networks of individuals with depression often exhibit reduced small-worldness, indicating a decline in local efficiency of functional connectivity, along with a weakening of global connectivity (19, 20). These changes in network structure may underlie the neural basis for emotional regulation and cognitive dysfunction.

This study aims to investigate the neural representations of the frontal cortex using fNIRS technology to examine the relationship between depressive status and sleep disturbances in postpartum women. We hypothesize that sleep disorders may lead to abnormal functional connectivity in specific regions within the frontal cortex, such as the dorsolateral prefrontal cortex and the ventromedial prefrontal cortex, which have been implicated in emotional regulation and cognitive control according to prior studies (21, 22). By focusing on these regions, our research aims to provide a more robust foundation for understanding the neural mechanisms underlying postpartum depressive status and sleep disturbances, and to highlight key areas for future research.

## Methods

### Participant

A total of 201 postpartum women who underwent a physical examination 42 days after delivery at the Beijing Haidian Maternal and Child Health Hospital from June 2021 to January 2022 were initially included in the study. Their ages ranged from 22 to 43 years, with an average age of 30.06 ± 3.86 years.

Inclusion criteria: (1) Participants were included if they were 42 days post-delivery. The defined “postpartum” as the period starting immediately after childbirth and extending to 6 weeks after delivery. This period is consistent with the clinical definition of early postpartum. (2) Only women who had a singleton, live infant delivery were included in the study. (3) Participants were required to be permanent residents of Beijing, having lived in the city throughout pregnancy and within 1 year after childbirth. (4) All participants had to have clear consciousness and be capable of independently and accurately completing the questionnaires.

Exclusion criteria: (1) Participants with a history of psychiatric disorders (e.g., depression, anxiety, and bipolar disorder) were excluded to avoid confounding effects from pre-existing mental health conditions. (2) Women with any neurological diseases were excluded to ensure that the study focused on the mental health effects of postpartum conditions. (3) Complicated Pregnancies or Medical Conditions: Those with medical conditions that could contribute to postpartum depression or affect sleep patterns (e.g., pre-eclampsia, hypothyroidism) were excluded from the study. (4) Drug Abuse or Other Substance Use Disorders: Participants with a history of drug or alcohol abuse were excluded due to their potential influence on mental health outcomes. (5) Women who had experienced multiple pregnancies (e.g., twins, triplets) were excluded to control for the possible additional physical and emotional strain of caring for more than one infant. (6) Participants who failed to provide complete questionnaire responses were excluded from the final analysis.

All participants signed informed consent voluntarily before the study began, and the research was approved by the Ethics Committee of the Department of Psychological and Cognitive Sciences at Tsinghua University (Protocol Number: 60101).

### Diagnostic criteria for sleep disorders

Insomnia is diagnosed by clinicians according to the criteria in the Chinese Guidelines for the Diagnosis and Treatment of Adult Insomnia (23), including: (1) The latency period of falling asleep is prolonged, and the sleep time is >30 min; (2) Sleep maintenance disorder, wake up more than twice during the night or wake up early; (3) Decreased sleep quality; (4) Reduced total sleep time, usually <6 h.

### Diagnostic criteria for postpartum depression

The Chinese version of the Edinburgh Postnatal Depression Scale (EPDS) was used to screen for postpartum depressive status (24), which is a self-report questionnaire consisting of 10 items with a total score of 30 points. It has been proven to have sufficient reliability and validity (25) and is widely used in clinical and research settings within Chinese-speaking populations (26, 27). In this study, a total score of 10 points was chosen as the critical value for screening postpartum depression, with a sensitivity and specificity of 85 and 84% (28). The survey was conducted using the Wenjuanxing platform (wjx.cn) on mobile phones, ensuring the authenticity and effectiveness of the questionnaire results.

### Verbal fluency task

**Figure 1:**
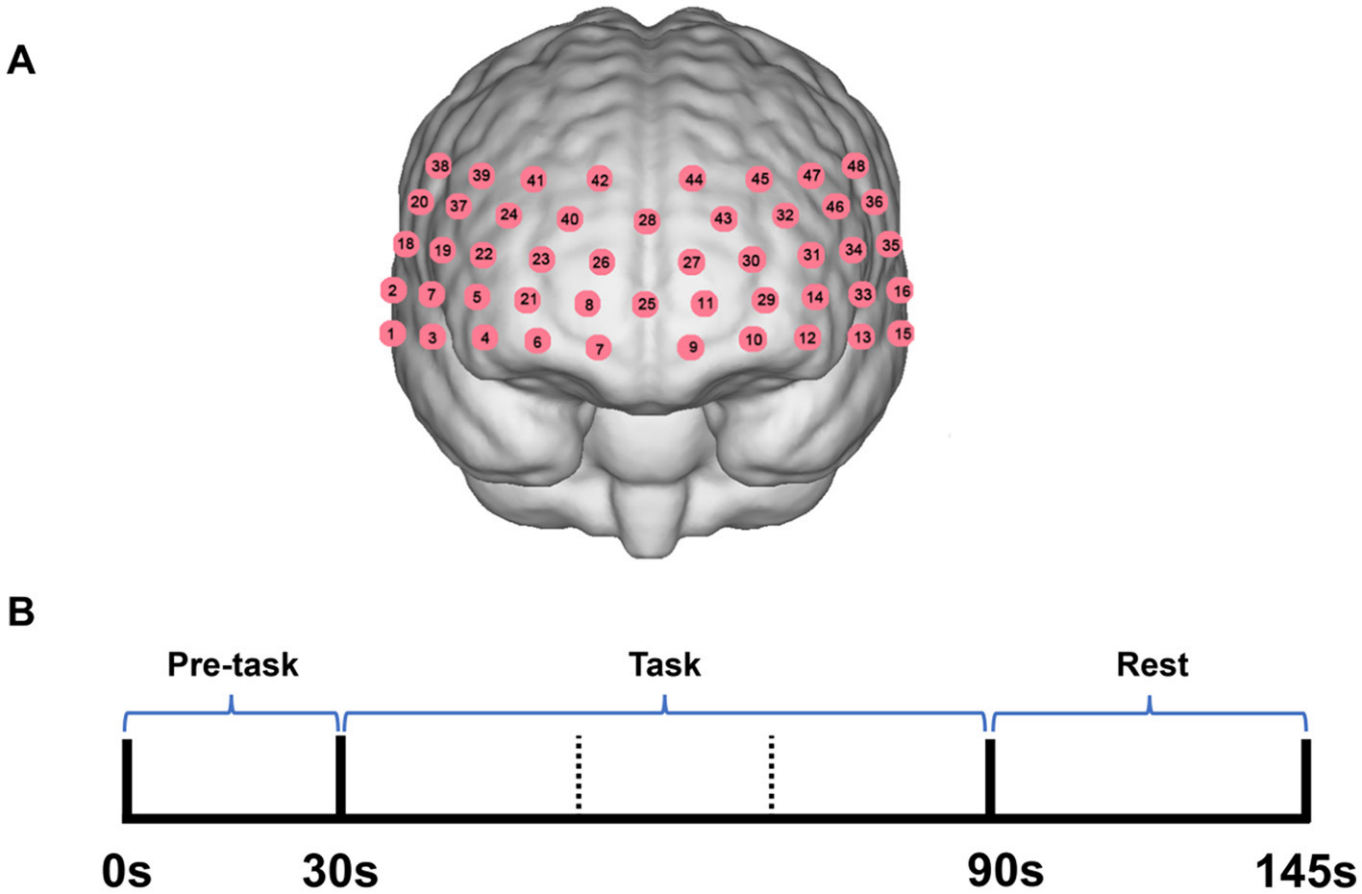
**(A)** The position of channels. **(B)** The verbal fluency test used for the functional near-infrared spectroscopy (fNIRS). Participants started saying “one,” “two,” “three,” “four,” and “five” continuously for 30 s in Chinese at pre-task. During the task period, participants were asked to list as many items as possible under the categories of the color or fruits in 60 s.

The verbal fluency task (VFT) was applied to assess vocabulary, lexical access speed, and executive function (29). In this study, the Chinese version of VFT was used with three parts. During the first part (pre-task), participants started saying “one,” “two,” “three,” “four,” and “five” continuously for 30 s in Chinese. During the second parts (task period), participants were asked to list as many items as possible under the categories of the coloer or fruits in 60 s (Figure 1B). The total runtime was 145 s. Participants were introduced to the VFT to confirm their understanding of the instructions. It was ensured that participants could listen to voice prompts, could form Chinese words, and could articulate the results. During the assessments, participants were seated in a comfortable and natural position, facing away from the screen. Their hands were placed naturally on their knees or the chair's armrest.

### NIRS measurement

Hemoglobin concentration was measured using a 48-channel near-infrared optical imaging system (NirScan, Danyang Huichuang Medical Equipment Co., Ltd., China). The placement of channels was focused on the anterior part of the brain, particularly the frontal and temporal regions. This choice was based on previous research that highlights the significance of these areas in understanding the neural mechanisms underlying postpartum depression and sleep disorder (30, 31). The sampling frequency was 11 Hz, and the wavelengths were 730, 808, and 850–730 and 850 nm were the major wavelengths and 808 nm was used as the isotopic wavelength for correction. The FPz channel in the 10/20 international system was used as the central midline probe. A total of 31 source-detector (SD) probes were placed, including 15 sources and 16 detectors. The probes were fixed at 3 cm intervals, covering the PFC of each subject, with the lowest probe placed along the Fp1-Fp2 line (Figure 1A). The channels and their corresponding brain regions are detailed in Supplementary Table 1.

### Data processing and analysis

The HOMER2 toolbox was employed, a graphical user interface program based on MATLAB 2013b (MathWorks, Inc.; Natick, US), for preprocessing the near-infrared spectroscopy data (32). Initially, a method based on motion standard deviation and spline interpolation was used to eliminate motion artifacts (33, 34). Motion artifacts were distinguished by identifying sliding window standard deviations that exceeded a certain threshold. Any signal changes in the data channels that exceeded the threshold (standard deviation, STD, and amplitude, AMP) were labeled as motion artifacts (hmr Motion Artifact By Channel; input parameters: tMotion = 0.5, tMask = 1, STD thresh = 30, AMPthresh = 0.5). Spline interpolation was applied to remove the artifacts (utilizing the hmr Motion Correct Spline function with input parameter *P* = 0.99). Subsequently, a second-order Butterworth bandpass filter with a cutoff frequency of 0.01–0.1 Hz was employed to remove physiological noise caused by heartbeats (1 Hz), respiration (~0.2–0.5 Hz), and high-frequency noise (35, 36). Finally, the filtered optical data was transformed into HbO and HbR concentrations using the modified Beer-Lambert law (37). This study focused on the HbO signal due to its ability to better reflect cortical activity (38). A 60-s task period was utilized as the time window for analyzing changes in oxygenated hemoglobin.

### Brain functional network construction and graph theory analysis

In this study, our objective is to analyze the brain functional network properties by extracting the time series of interest from all channels using photodetectors. Subsequently, Pearson correlation analysis was performed on these time series to obtain the correlation coefficient *r* between each pair of brain regions, generating the corresponding correlation coefficient matrix *R*_*ij*_. A specific threshold was then applied to the correlation matrix to binarize it, yielding the binarized connectivity matrix *R*_*ij*_ of the brain functional network. Since network parameters vary with the level of network sparsity and there is currently no standardized threshold for selecting the optimal sparsity level, we selected a set of sparsity thresholds with a step size of 0.01, ranging from a minimum of 0.1 to a maximum of 0.40 (0.1 < S < 0.40), based on previous experience (39). This resulted in a series of brain functional network sets with gradually increasing sparsity levels for each subject.

The brain functional properties calculated include the clustering coefficient, characteristic path length, global efficiency, and local efficiency, which were quantitatively measured using graph theory methods (40). The specific calculation formulas are as follows:

(1) The average of the shortest paths between all the two points in the network is defined as the average shortest path, which can usually be used to measure the integration of the network.
$$\begin{array}{l} L=\frac{1}{n}\underset{i\in N}{\sum }L_{i}=\frac{1}{n}\underset{i\in N}{\sum }\frac{\sum_{j\in N,j\neq i}d_{ij}^{-1}}{n-1} \end{array}$$
  where *L*_*i*_ is the average distance between a node and other nodes, *d*_*ij*_ represents the shortest path of a node.
(2) The clustering coefficient describes the clustering degree of nodes in a network, which measures the local information transmission ability of the network and the grouping degree of the network and also reflects the clustering characteristics of a network in a specific case.
$$\begin{array}{l} Cc=\frac{1}{n}\underset{i\in N}{\sum }C_{i}=\frac{1}{n}\underset{i\in N}{\sum }\frac{\sum_{j,h\in N}a_{ij}a_{ih}a_{jh}}{k_{i}\left(k_{i}-1\right)} \end{array}$$
  where *C*_*i*_ is the clustering coefficient of a node, *a*_*ij*_ represents the connection between nodes *i* and *j*, and *k*_*i*_ is the degree of a node.
(3) The global efficiency of the network is defined as the average of the reciprocal shortest paths of all nodes in the network, which is an important indicator to measure the speed of information transmission in the network, namely:
$$\begin{array}{l} E_{global}=\frac{1}{n}\underset{i\in N}{\sum }E_{i}=\frac{1}{n}\underset{i\in N}{\sum }\frac{\sum_{j\in N,j\neq i}d_{ij}^{-1}}{n-1} \end{array}$$
  where *E*_*i*_ is the efficiency of a node and *d*_*ij*_ represents the shortest path of the node.
(4) The local efficiency of a network represents the efficiency of information exchange between network nodes and sub-networks and can represent the working efficiency of the measured range. It is defined as:
$$\begin{array}{l} E_{local}=\frac{1}{n}\underset{i\in N}{\sum }E_{loc,i}=\frac{1}{n}\underset{i\in N}{\sum }\frac{\sum_{j\in N,j\neq i}a_{ij}a_{ih}{\left[d_{jh}\left(N_{i}\right)\right]}^{-1}}{k_{i}\left(k_{i}-1\right)} \end{array}$$
  where *E*_*loc,i*_ is the local efficiency of a node i and *d*_*jh*_(*N*_*i*_) is the shortest path length among all paths of node j and h passing through node *i*.
(5) Normalizing the topological parameters of a real network is often necessary to evaluate small-world characteristics. A common method is to reference matched random networks, comparing the topological parameters of the real network with those of the corresponding random networks. In this study, we employed the network randomization method proposed by Maslov and Sneppen in 2002. We constructed 100 random networks with the same number of nodes, edges, and degree distribution as the real network. We calculated the average values of the topological parameters (Cp_rand and Lp_rand) for these 100 random networks. Normalized small-world topological indices were obtained by computing the ratio of the real network's topological parameters to the average values of the corresponding random network's topological parameters.

If a real network's normalized topological indices meet the criteria ${\gamma =C}_{p}^{norm\;}=C_{p_{-}real\;}/C_{p_{-}rand\;}>1$ and $\lambda =L_{p}^{norm\;}=L_{p_{-}real\;}/L_{p_{-}rand\;}\approx 1$, it is considered to exhibit small-world topological organization. Additionally, a comprehensive measure of small-world network characteristics, denoted as σ = γ/λ > 1, can be defined.

### Statistical analyses

Statistical analysis was conducted using SPSS 20.0 (IBM Corp., NY, USA). Graphs were generated using the NirsKit package and GraphPad Prism 8 software. The Shapiro-Wilk test was applied to assess the normality of the data. Continuous variables were expressed as means ± standard deviations, and categorical variables were expressed as numbers (percentages). Demographic data were analyzed using independent sample *t*-tests. Functional connectivity was examined by Pearson correlation analysis of the time series of each channel pair. Brain network topological properties, including small-world attributes, clustering coefficient, global efficiency, local efficiency, and characteristic path length, were compared between groups using independent sample *t*-tests or Mann-Whitney tests. The relationship between postpartum women's sleep status and EPDS scores was evaluated using Spearman's correlation. Statistical significance was set at *p* < 0.05; all *p*-values were two-tailed. False discovery rate FDR correction was applied to results from multiple comparisons across channels.

## Results

### Demographic and clinical characteristics

Out of the initial screening, a total of 58 participants were excluded for the following reasons: six due to pre-existing medical conditions, seven due to pre-existing psychiatric conditions, 14 due to multiple pregnancies, and 31 due to incomplete data. As a result, 143 postpartum women were included in the final analysis. The sleep status of 143 postpartum women was assessed by clinicians. Forty of them were included in the insomnia group with a positivity rate of 27.97 % (mean age = 33.63 years; SD = 3.43), while 103 without insomnia were included in the control group (mean age = 31.20 years; SD = 3.40). The depression status of 143 postpartum women was also assessed using EPDS ≥10 points as a threshold. The EPDS scores were significantly higher in the insomnia group than in the control group (Mann-Whitney test: *Z* = 2.71, *p* = 0.007). Of the 40 postpartum women with insomnia, eight had an EPDS score ≥ 10. Among the 103 postpartum women without insomnia, six had an EPDS score ≥ 10.

### Brain functional connectivity of PFC

**Figure 2:**
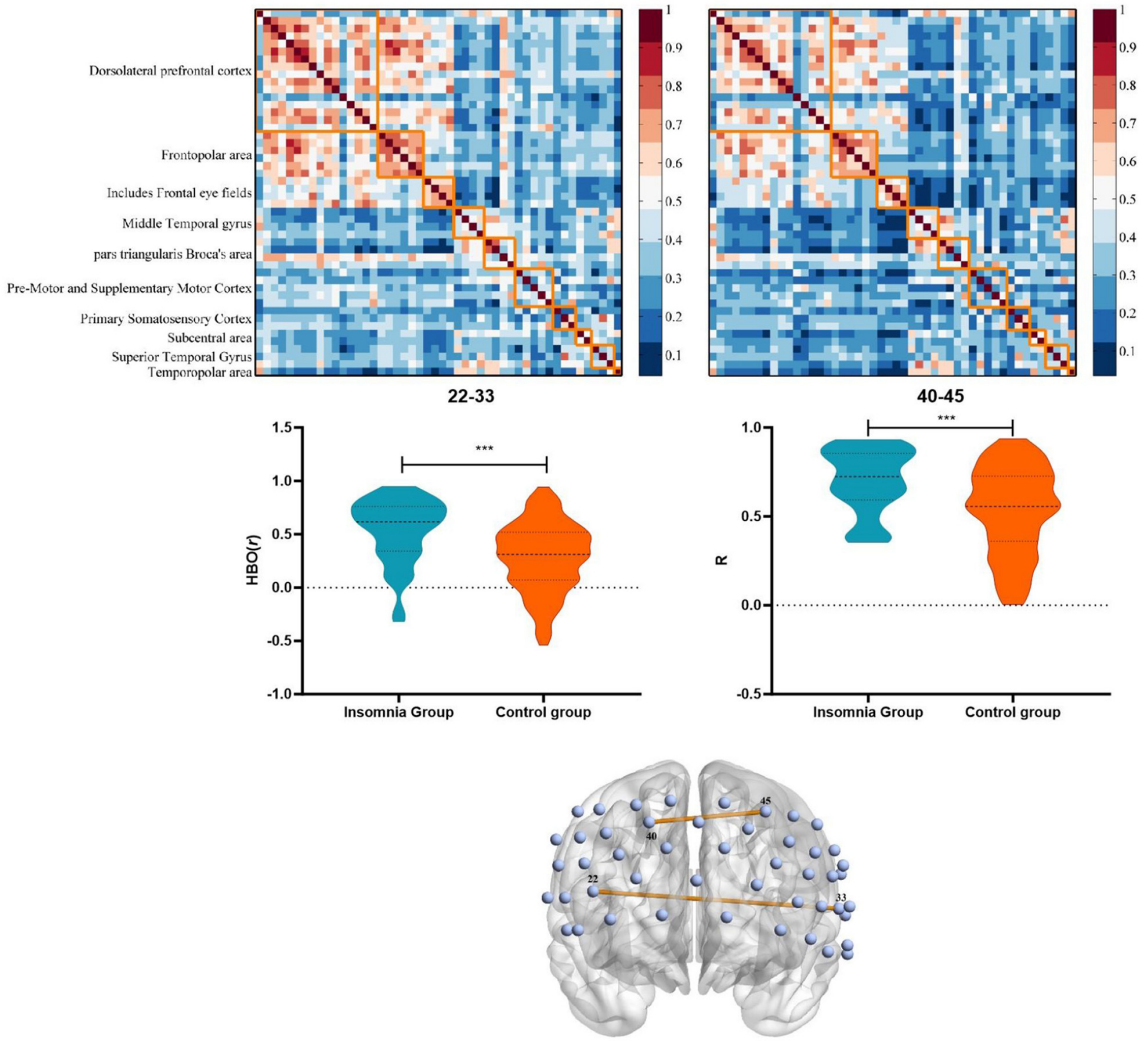
Characteristics and differences of brain functional connectivity between insomnia group and control group based on HbO. ***p < 0.001.

We examined the functional connectivity strength of the whole PFC based on HbO signals. Two 48 × 48 connectivity coefficient matrix of the functional brain network for insomnia and control group were calculated (Figure 2). The results showed that the 22–33 [HbO: (0.53 ± 0.31 vs. 0.28 ± 0.32), *t*_(141)_ = −4.20, *p* < 0.001, 95% CL (0.13–0.37)] and the 40–45 [HbO: (0.70 ± 0.18 vs. 0.53 ± 0.25), *t*_(141)_ = −3.97, *p* < 0.001, 95% CL (0.09–0.26)] channel pairs connection were significantly higher in the insomnia group than in the control group, which passed the FDR correction. The differences in brain network connectivity are shown in Figure 2. Channel 22 represents pars triangularis Broca's area, and 33 represents Middle and Superior Temporal gyrus; Channels 40 and 45 represent the left and right dorsolateral prefrontal cortex (dlPFC), respectively (Supplementary Table 1).

### Network topological properties

**Figure 3:**
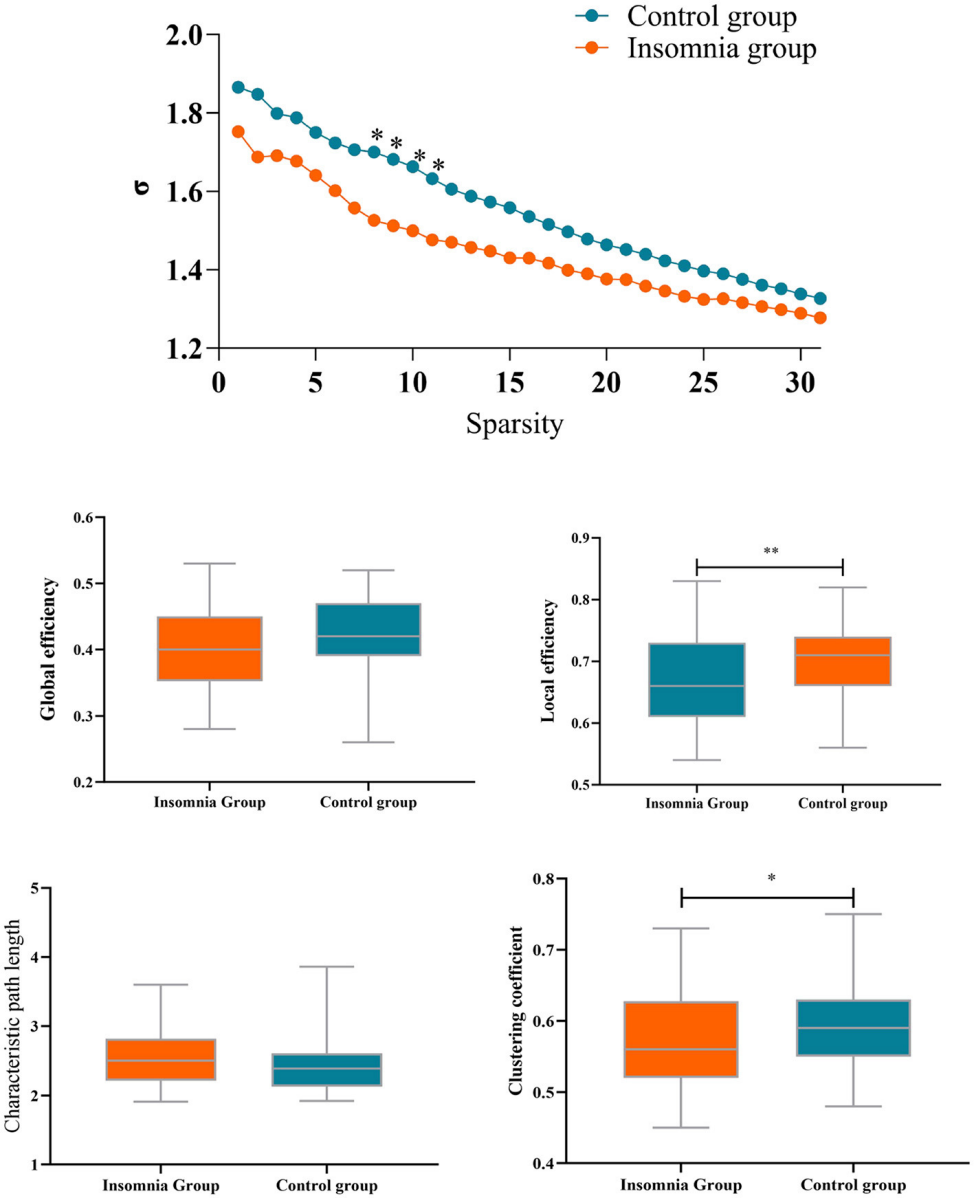
Network metrics in a range of sparsity threshold. *p < 0.05; **p < 0.01.

As depicted in Figure 3, small-world topology properties, quantified by the σ metric, were >1 in both groups across a wide range of sparsity thresholds (0.1 < S < 0.4). However, lower small-world topology metrics were observed in the insomnia group compared to the healthy group. Significant differences were observed in the sparsity thresholds of 0.17, 0.18, 0.19, and 0.20. Consequently, small-world topology metrics were analyzed within the sparsity range with significant differences between the insomnia group and the control group.

Compared with the control group, the insomnia group had a significantly lower clustering coefficient (*p* = 0.009, mean difference = 0.030) and local efficiency (*p* = 0.020, mean difference = 0.025) of brain functional networks, whereas no significant difference was found in global efficiency (*p* = 0.13, mean difference = 0.017) and characteristic path length (*p* = 0.12, mean difference = 0.125). Next, the network topology properties are computed for each node. Results revealed that only node 12 (left temporal pole) exhibited significantly reduced local efficiency (*p* < 0.000, mean difference = 0.259) and clustering coefficient (*p* < 0.000, mean difference = 0.225) compared to the control group. Differential *p*-values for brain regions are presented in Figure 3.

### Correlation analysis result

**Figure 4:**
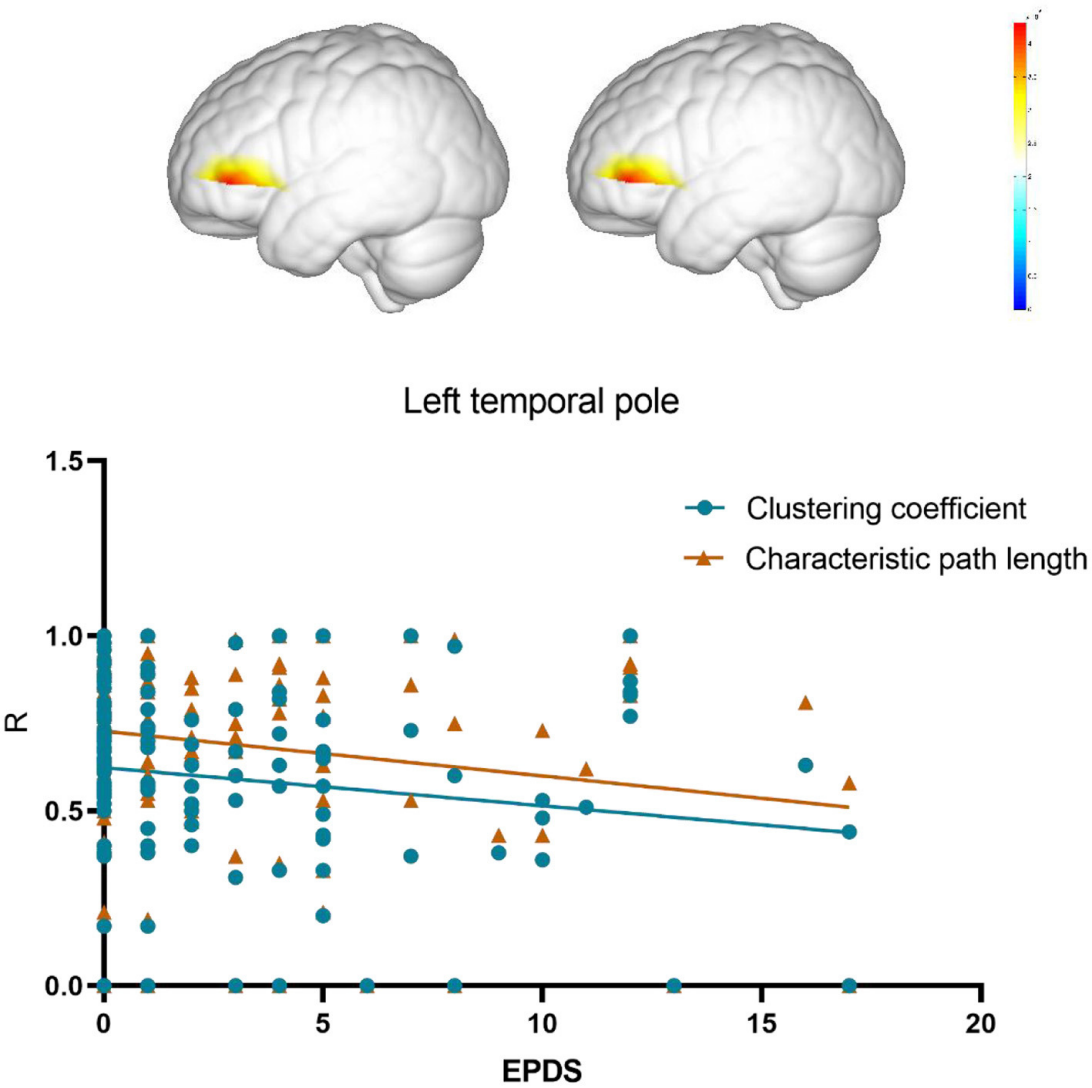
Correlation between EPDS and functional connectivity coefficient of brain.

To further confirm the association between changes in network topology properties of the temporal pole area induced by insomnia and postpartum depression, the Spearman correlation analysis was conducted to analysis between the local efficiency and clustering coefficient of the left temporal pole and EPDS score. The results revealed a negative correlation between the local efficiency of the temporal pole area and EPDS scores in the postpartum woman (*r* = −0.19, *p* = 0.03). Also, the characteristic path length of the temporal pole area exhibited a negative correlation with EPDS scores (*r* = −0.2, *p* = 0.02; Figure 4).

## Discussion

This study investigated the relationship between insomnia and depressive symptoms in postpartum women, with a focus on examining whether changes in brain functional network properties within the frontal lobe could serve as a potential mechanism linking sleep issues to depression. Using fNIRS technology, we analyzed brain functional connectivity and topological features in postpartum women. The results showed significantly increased functional connectivity between the right and left dorsolateral prefrontal cortex in the insomnia group. Additionally, increased connectivity was found between the right Broca's area and the left middle temporal gyrus. Further analyses demonstrated that postpartum women with insomnia exhibited reduced local efficiency and clustering coefficient of brain networks, particularly in the temporal pole region. Notably, the local efficiency and clustering coefficient reductions in this region were significantly associated with higher EPDS scores, indicating a relationship with depressive symptoms. These findings suggest that alterations in brain network properties, particularly in the temporal pole, may play a role in the development of depressive symptoms associated with insomnia in the postpartum period.

### Brain functional network characteristics

Our study results reveal the EPDS scores of insomnia group were significantly higher than those in the control group, indicating a close association between insomnia and postpartum depressive states. This finding is consistent with previous research, supporting the link between sleep disorders and postpartum depression (41, 42).

The brain is considered to be a dynamic and interconnected functional network. Sleep is crucial in maintaining normal brain function, and global or local dysfunction may lead to insomnia. The PFC is involved in maintaining the quiescent state of the brain, integrating internal and external environmental information, emotion regulation, and episodic memory retrieval (43). Researchers have used different brain imaging techniques, such as fMRI, EEG, and fNIRS, to explore the PFC function in patients with chronic insomnia disorder. The fMRI study by Altena et al. (44) revealed low activation of medial and inferior frontal cortex regions in insomnia patients, highlighting the importance of the PFC in sleep. An EEG study by Perrier et al. (45) found a lower power spectrum of β1 in the prefrontal cortex in patients with primary insomnia. The fNIRS study by Gong et al. (46) showed a significant reduction in prefrontal mean channel functional connectivity in patients with insomnia.

The connectivity between the left and right dlPFC was enhanced in the insomnia group compared with the control group. The dlPFC is a region closely associated with cognitive functions such as emotion regulation, cognitive control, and attention (47). The enhanced connectivity between the dlPFC may reflect an increased information transfer and functional coordination between these brain regions in postpartum women with insomnia. Women in the postpartum period experience dramatic changes in their lives, including the care of a newborn and changes in life patterns. These factors may have led to decreased sleep quality and triggered enhanced prefrontal activity in emotional and cognitive control in response to these challenges (41, 48). This alteration of the functional brain network may be intended to stabilize cognitive functioning during sleep-disordered states.

The female brain undergoes dynamic neural plasticity during pregnancy and the postpartum period, with notable gray matter volume reductions in various brain regions, such as the hippocampus, cingulate cortex, medial orbital frontal cortex, and insula (49, 50). These changes have been shown to play critical roles in social processes (51), emotion regulation (52), and their relevance to the development of depression (53). The increased connectivity in the dorsolateral prefrontal cortex, a region crucial for emotion regulation and cognitive control, may signify adaptive alterations in postpartum women with sleep disorders in handling emotional and attention aspects. While these adaptations are believed to prepare new mothers for their roles, they cannot rule out the potential contributions to the occurrence of psychiatric disorders (54), given the concurrent nature of structural brain changes and postpartum mental health issues. Significantly higher EPDS scores in the sleep-disordered group further support this point.

The results also revealed enhanced connectivity between the right Broca's area and the left superior temporal gyrus, regions closely associated with cognitive functions such as language processing and semantic comprehension. This study involved activating brain areas related to language processing during the VFT. Thus, the increased connectivity in postpartum women with insomnia may reflect intensified information transmission and functional coordination between different brain hemispheres. Borragán et al. (55) found that in normal individuals staying awake for a short period, the right PFC exhibited increased activity, while the left PFC showed reduced activity. Furthermore, Honma et al. (56) demonstrated that in alertness tests for healthy subjects, increased activation in the right prefrontal cortex was positively correlated with alertness. This suggests that activating the right prefrontal cortex may assist healthy individuals or short-term insomniacs overcome drowsiness and provide sufficient activity to meet the demands of more cognitively challenging tasks.

In short, insomnia have a notable impact on the PFC in postpartum women. Postpartum women may adapt to effects by altering connectivity between brain regions to maintain stable cognitive and emotional functions. This additional enhancement of functional connectivity might serve as a compensatory mechanism to address potential deficiencies in other brain regions. The intensified connectivity could be a natural physiological response, but it may also have implications for sleep problems and the risk of depression. However, further research is needed to gain a deeper understanding of these intricate relationships, determining whether enhanced prefrontal cortex activity plays a positive or negative role and how it influences sleep and depressive states.

### The topology properties of the brain functional network

The topological properties of prefrontal brain networks were analyzed based on graph theory to understand the mechanisms underlying the adjustment of functional brain networks between the insomnia group and the control group. Small-world attributes are crucial indicators describing network connectivity and information transmission efficiency in brain networks. Small-world networks exhibit a balance of high clustering and short path lengths, meaning that nodes in the network tend to form tight clusters and are interconnected through relatively short pathways (57). In the postpartum insomnia group, the brain functional network maintains a small-world topology similar to healthy participants, suggesting a balance between local specialization and global integration in information processing remains (58). However, the small-world topology indices in postpartum women with sleep disorders were lower than in the healthy control group. The reduced small-world attributes might reflect abnormalities in functional connectivity and integration in the prefrontal cortex region (58). This implies that information transmission and integration in the prefrontal cortex region could be disrupted in the context of sleep disorders, showing significant differences in the network's sparsity range from 0.17 to 0.20.

Further analysis revealed that in postpartum women with sleep disorders, local efficiency and clustering coefficient were significantly reduced, while network global efficiency and characteristic path length did not exhibit significant reductions. Local efficiency is an indicator describing the efficiency of information transmission between nodes in a network, with higher local efficiency indicating that nodes in the network are more efficient in information transmission and integration (59). The clustering coefficient measures the degree to which nodes in the network cluster together, representing the tendency of nodes in the network to form tightly-knit clusters (59). Local efficiency and clustering coefficient reflect the local information processing capability of nodes in the network and the degree of close connections between nodes.

The reduction in local efficiency and clustering coefficient may suggest that in postpartum women with sleep disorders, there is a decrease in the brain's ability to transmit and process local information efficiently. Further analysis of the local efficiency and clustering coefficient for each node showed a significant decrease in these measures in the temporal pole region of the insomnia group. The temporal pole is closely associated with various functions, including memory, emotions, language, and spatial cognition (60). The reduction in local efficiency and clustering coefficient may imply a functional decline in the temporal pole region of postpartum women with sleep disorders, which could decrease their ability to process and regulate information, affecting aspects such as memory, emotions, and language capabilities. The reduction in local efficiency and clustering coefficient may also reflect a decrease in the connectivity and integration capabilities between the temporal pole region and other brain areas. Impaired information transmission and coordination between the temporal pole region and other areas of the brain could lead to isolation and disrupted information transfer in certain functional areas of the network, thereby affecting overall information processing and functional integration. The reduced local efficiency and clustering coefficient of the temporal pole nodes were negatively correlated with depressive symptoms, and previous research has already shown the crucial role of the temporal pole in emotion regulation and cognitive function (61). Functional abnormalities in the temporal pole region may lead to difficulties in emotion regulation and cognitive processing in postpartum women, associated with the onset and persistence of depressive symptoms. While our results suggest a potential link between functional brain network alterations in the temporal pole area and EPDS scores, these findings remain exploratory due to the relatively small sample size. Future research with larger cohorts is essential to validate these associations and establish stronger evidence for the role of functional connectivity changes in postpartum depression and sleep disorders.

Future studies should address the limitations identified in our current analysis by employing a more granular approach to patient classification. Specifically, increasing sample sizes and conducting detailed subgroup analyses—differentiating between patients with sleep disorders and depression, those with sleep disorders without depression, patients with depression without sleep disorders, and those with neither condition—will allow researchers to better isolate the independent and combined effects of sleep disturbances and depression on brain connectivity. This refined methodology is expected to enhance the robustness of findings and lead to more specific conclusions, ultimately improving the generalizability of results to a broader population of postpartum patients. Moreover, incorporating longitudinal designs could provide valuable insights into how these relationships evolve over time, thereby deepening our understanding of the interplay between sleep and mental health in postpartum contexts. To further strengthen future research, we recommend including a detailed assessment of support systems, such as caregiving duties, the presence of family or spouses, and the use of external childcare. This will help to elucidate additional factors that may influence the mental health and sleep quality of postpartum individuals. This study did not include NIRS measurements both pre- and post-partum. While the inclusion of such measurements could provide valuable insights into the neural changes associated with postpartum depression, the constraints of this design limited our ability to implement them. Future research should consider incorporating pre- and post-partum comparisons to enhance understanding of brain activity changes during this critical period. Future studies could incorporate neuroimaging techniques like MRI to examine potential volumetric differences alongside functional assessments, offering a more comprehensive view of the neurobiological mechanisms involved in postpartum sleep disorders.

## Conclusion

This study highlights a potential link between insomnia and depressive symptoms in postpartum women, with initial findings suggesting that alterations in brain network properties may play a role in this relationship. Specifically, changes in local efficiency and clustering coefficients were observed in areas associated with emotional regulation. However, it is important to note that these findings are preliminary, and definitive conclusions about the mechanisms by which sleep disturbances influence depressive symptoms cannot be made at this stage. Further research is needed to clarify these relationships and to explore the role of additional neural networks in postpartum depression.

## Data Availability

The raw data supporting the conclusions of this article will be made available by the authors, without undue reservation.
