# Supplementary material for: The impact of sleep disorders on brain network connectivity in postpartum women: a functional near-infrared spectroscopy-based study
Source: Front Neurol. 2024 Dec 12;15:1487985. doi: 10.3389/fneur.2024.1487985 (PMC11669591; doi:10.3389/fneur.2024.1487985)
Supplement: Supplementary file 1 [file Table_1.docx]

**Supplementary Materials**

**1 The location information of all channels in the region of interest (ROI)**

The location information of all channels covering Montreal Neurological Institute (MNI) coordinates and the corresponding Brodmann Area (BA) was listed in the following table.

**Table S1 The location information of all channels**

| CH | Brodmann Area (Percentage of Overlap %) | MNI | | |
| --- | --- | --- | --- | --- |
|  |  | X | Y | Z |
| CH1 (S1-D1) | 21 - Middle Temporal gyrus (97) | -71 | 12 | -6 |
| CH2 (S1-D6) | 21 - Middle Temporal gyrus (61) | -72 | 27 | 12 |
| CH3 (S2-D1) | 38 - Temporopolar area (78) | 59 | 18 | 1 |
| CH4 (S2-D2) | 47 - Inferior prefrontal gyrus (43) | 54 | 45 | 1 |
| CH5 (S2-D7) | 45 - pars triangularis Broca's area (54) | 60 | 29 | 16 |
| CH6 (S3-D2) | 47 - Inferior prefrontal gyrus (32) | 39 | 64 | 6 |
| CH7 (S3-D3) | 11 - Orbitofrontal area (84) | 15 | 74 | 8 |
| CH8 (S3-D8) | 10 - Frontopolar area (55) | 27 | 65 | 25 |
| CH9 (S4-D3) | 11 - Orbitofrontal area (99) | -15 | 73 | 7 |
| CH10 (S4-D4) | 46 - Dorsolateral prefrontal cortex (52) | -40 | 61 | 1 |
| CH11 (S4-D9) | 11 - Orbitofrontal area (57) | -29 | 63 | 22 |
| CH12 (S5-D4) | 38 - Temporopolar area (99) | -54 | 40 | -3 |
| CH13 (S5-D5) | 21 - Middle Temporal gyrus (98) | -62 | 5 | -9 |
| CH14 (S5-D10) | 38 - Temporopolar area (62) | -59 | 23 | 12 |
| CH15 (S6-D5) | 21 - Middle Temporal gyrus (85) | -71 | -19 | -10 |
| CH16 (S6-D11) | 21 - Middle Temporal gyrus (67) | -70 | -34 | 8 |
| CH17 (S7-D1) | 48 - Retrosubicular area (59) | 68 | 1 | 16 |
| CH18 (S7-D6) | 22 - Superior Temporal Gyrus (53) | -69 | 14 | 31 |
| CH19 (S7-D7) | 6 - Pre-Motor and Supplementary Motor Cortex (43) | 63 | 11 | 31 |
| CH20 (S7-D12) | 43 - Subcentral area (56) | 64 | -9 | 43 |
| CH21 (S8-D2) | 46 - Dorsolateral prefrontal cortex (85) | 47 | 51 | 19 |
| CH22 (S8-D7) | 45 - pars triangularis Broca's area (98) | 51 | 34 | 32 |
| CH23 (S8-D8) | 46 - Dorsolateral prefrontal cortex (56) | 35 | 50 | 36 |
| CH24 (S8-D13) | 45 - pars triangularis Broca's area (80) | 41 | 33 | 46 |
| CH25 (S9-D3) | 10 - Frontopolar area (88) | -1 | 65 | 24 |
| CH26 (S9-D8) | 10 - Frontopolar area (100) | 13 | 59 | 39 |
| CH27 (S9-D9) | 10 - Frontopolar area (100) | -14 | 58 | 39 |
| CH28 (S9-D14) | 10 - Frontopolar area (100) | -2 | 47 | 51 |
| CH29 (S10-D4) | 46 - Dorsolateral prefrontal cortex (67) | -48 | 47 | 14 |
| CH30 (S10-D9) | 46 - Dorsolateral prefrontal cortex (61) | -38 | 48 | 32 |
| CH31 (S10-D10) | 45 - pars triangularis Broca's area (100) | -53 | 30 | 27 |
| CH32 (S10-D15) | 45 - pars triangularis Broca's area (90) | -44 | 30 | 42 |
| CH33 (S11-D5) | 21 - Middle Temporal gyrus (54) | -67 | -6 | 11 |
| CH34 (S11-D10) | 48 - Retrosubicular area (58) | -64 | 5 | 26 |
| CH35 (S11-D11) | 22 - Superior Temporal Gyrus (80) | -68 | -20 | 27 |
| CH36 (S11-D16) | 43 - Subcentral area (72) | -65 | -14 | 38 |
| CH37 (S12-D7) | 45 - pars triangularis Broca's area (58) | 53 | 15 | 44 |
| CH38 (S12-D12) | 6 - Pre-Motor and Supplementary Motor Cortex (62) | 54 | -5 | 56 |
| CH39 (S12-D13) | 45 - pars triangularis Broca's area (49) | 44 | 16 | 57 |
| CH40 (S13-D8) | 46 - Dorsolateral prefrontal cortex (57) | 21 | 44 | 51 |
| CH41 (S13-D13) | 46 - Dorsolateral prefrontal cortex (53) | 27 | 27 | 59 |
| CH42 (S13-D14) | 9 - Dorsolateral prefrontal cortex (86) | 11 | 34 | 61 |
| CH43 (S14-D9) | 46 - Dorsolateral prefrontal cortex (55) | -25 | 43 | 48 |
| CH44 (S14-D14) | 9 - Dorsolateral prefrontal cortex (90) | -14 | 33 | 60 |
| CH45 (S14-D15) | 46 - Dorsolateral prefrontal cortex (57) | -33 | 25 | 56 |
| CH46 (S15-D10) | 44 - pars opercularis_ part of Broca's area (60) | -56 | 11 | 37 |
| CH47 (S15-D15) | 44 - pars opercularis_ part of Broca's area (52) | -46 | 14 | 54 |
| CH48 (S15-D16) | 6 - Pre-Motor and Supplementary Motor Cortex (55) | -57 | -10 | 50 |
